# Plastamination: A One Health and Planetary Health Perspective on a Rising Global Crisis

**DOI:** 10.7759/cureus.87523

**Published:** 2025-07-08

**Authors:** Abdulqadir J Nashwan, Kalpana Singh

**Affiliations:** 1 Nursing and Midwifery Research, Hamad Medical Corporation, Doha, QAT

**Keywords:** climate change, environmental exposure, microplastics, nanoplastics, neurotoxicity, one health, planetary health, plastic pollution, soil microbiota, sustainable materials

## Abstract

Plastamination, the pervasive integration of microplastics and nanoplastics into ecological and biological systems, represents a growing yet underrecognized environmental health crisis. This editorial explores the systemic consequences of plastic particle exposure on human health, including neurotoxicity, mitochondrial dysfunction, and gut microbiota disruption, alongside its ecological impacts on soil fertility, food security, and climate change. Drawing on the One Health and planetary health frameworks, the piece calls for urgent regulatory, scientific, and policy interventions to mitigate plastamination’s far-reaching risks and ensure intergenerational environmental justice.

## Editorial

Plastamination, the persistent embedding of microplastics (MPs) and nanoplastics (NPs) in biological and ecological systems, is an emerging and hidden environmental threat with significant implications for One Health and planetary health. Sometimes used interchangeably, both emphasize that human well-being is deeply interconnected with the health of animals, ecosystems, and the planet, underscoring the need for holistic approaches to safeguard all life [1]. MPs are generally defined as plastic particles less than 5 mm in diameter, while NPs are typically smaller than 1 μm in size [1]. These size distinctions are crucial as they influence the environmental behavior, biological interactions, and toxicological profiles of plastic particles. While global attention often focuses on the visible burden of plastic waste, the subtle and far-reaching consequences of MPs/NPs infiltration remain underrecognized.

The One Health and Planetary Health frameworks provide critical lenses through which the systemic risks of plastamination can be understood and addressed. The One Health approach emphasizes the deep interdependence between human, animal, and environmental health, underscoring how plastic pollution in one domain can have a ripple effect across others [1]. For instance, ingestion of plastic particles by marine organisms not only disrupts biodiversity but also introduces toxicants into the human food chain, while MPs in soil impair agricultural productivity and ultimately affect food security. Complementing this, the Planetary Health framework situates plastamination within broader ecological and sociopolitical systems, recognizing that human well-being is inseparable from the stability of Earth’s natural systems. Plastamination exemplifies how the breach of planetary boundaries - through unchecked plastic production, pollution, and inadequate waste governance - can erode ecosystem resilience, amplify health disparities, and undermine climate action. Together, these frameworks call for integrated, cross-sectoral responses that prioritize sustainability, intergenerational justice, and global cooperation in safeguarding health at all levels of life.

Global plastic production has surged from two million tons in 1950 to over 460 million tons annually by 2023, with projections exceeding one billion tons by 2060 if current trends persist [2,3]. Alarmingly, recent estimates indicate that at least 14 million tons of plastic enter the ocean annually, contributing to the accumulation of over 170 trillion plastic particles floating in marine environments [4]. Humans are estimated to ingest up to 5 g of plastic weekly, highlighting the ubiquity of this exposure [5]. MPs/NPs have been detected in human placentas, fetal brains, and neural tissue, where they may accumulate at higher levels than in organs such as the liver and kidneys [6,7]. Human exposure to MPs and NPs arises from multiple sources, including contaminated food and water, inhalation of airborne particles, and dermal contact with personal care products or synthetic textiles [8]. Common contributors include bottled water, seafood, processed foods, urban dust, and indoor air, especially in environments with high levels of synthetic materials or plastic degradation [8]. Despite increasing awareness of MP and NP exposure, substantial methodological challenges persist that hinder the accurate detection and quantification of these pollutants in human samples. Techniques such as micro-Fourier-transform infrared spectroscopy and Raman spectroscopy have been proposed for identifying MPs in human blood, placenta, and cerebrospinal fluid [9]. However, these methods require labor-intensive sample preparation, are prone to contamination, and are not yet standardized or widely implemented in clinical settings [9]. Consequently, current exposure estimates may under- or overrepresent actual body burdens.

Experimental studies have demonstrated that NPs can cross the blood-brain barrier (BBB), accumulate in neurons, and impair mitochondrial function, resulting in cellular energy deficits and apoptosis. These particles may promote the aggregation of pathological proteins such as α-synuclein, a hallmark of neurodegenerative diseases like Parkinson’s [10]. Emerging evidence underscores the neurotoxic and reproductive consequences of plastamination, as detailed by Santoro et al. (2024), who reviewed extensive in vitro and in vivo data revealing that both non-biodegradable and biodegradable MPs/NPs can cross biological barriers, accumulate in sensitive tissues, and trigger systemic dysfunction. Their findings highlight the capacity of MPs/NPs to induce oxidative stress, mitochondrial damage, and chronic inflammation in neural and gonadal tissues, thereby contributing to neuroinflammation, synaptic dysregulation, reduced gamete quality, and altered reproductive hormone signaling. These adverse effects, notably observed even at environmentally relevant exposure levels, raise concern over the long-term impacts on neurodevelopment, cognitive function, and fertility across generations [11]. However, these findings must be interpreted with caution. Many experimental studies employ high concentrations of NPs [12], which may not accurately reflect typical environmental exposure levels, thereby raising concerns about the ecological validity of these studies. Dose-response relationships and threshold effects for neurotoxicity, mitochondrial dysfunction, and systemic inflammation remain poorly characterized in humans [13]. Additionally, interspecies differences in barrier permeability, particle uptake, and immune responses complicate the extrapolation of animal data to human health outcomes. These limitations underscore the need for more robust epidemiological studies, human-relevant exposure models, and longitudinal designs to clarify real-world risks and inform evidence-based regulation.

Additionally, NPs have been shown to disrupt the composition and diversity of the gut microbiota, leading to microbial dysbiosis characterized by a decline in beneficial commensals and an increase in pro-inflammatory bacterial taxa [14]. This imbalance undermines intestinal barrier integrity, facilitating the translocation of microbial metabolites and endotoxins, such as lipopolysaccharide, into the systemic circulation and activating peripheral immune responses [14]. The resulting low-grade systemic inflammation extends to the central nervous system via the gut-brain axis, where it contributes to microglial activation, alterations in BBB permeability, and neuroinflammatory cascades [11]. These processes collectively exacerbate neural dysfunction and heighten the risk of neurodevelopmental and neurodegenerative disorders.

Beyond human health, plastamination poses grave risks to ecosystems. MPs reduce soil fertility by disrupting microbial communities essential for crop productivity, threatening food security and biodiversity. Studies have shown that MPs can alter the abundance of key microbial taxa involved in nitrogen fixation and organic matter decomposition, leading to reduced enzymatic activity and disrupted biogeochemical processes. These changes impair soil quality, limit crop productivity, and ultimately threaten food security and biodiversity [15,16]. In aquatic systems, MPs have been found to interfere with plankton reproduction, impair photosynthetic efficiency in algae, and accumulate in the tissues of fish and invertebrates, cascading up the food chain and altering ecosystem dynamics [17]. Moreover, the production of plastics is a significant contributor to climate change, responsible for substantial carbon emissions throughout its life cycle, from fossil fuel extraction and polymer synthesis to waste incineration, exacerbating planetary health challenges and undermining global efforts to achieve climate goals such as those outlined in the Paris Agreement [18,19].

The European Union’s plastic strategy promotes biodegradable plastics, such as polylactic acid (PLA), as alternatives to conventional plastics like polypropylene (PP) to mitigate environmental harm [20]. However, a recent study testing PLA and PP-MPs, as well as their leachates, on seven marine species across trophic levels revealed that both types of MPs caused toxic effects in three consumer species, particularly *Artemia franciscana*, with more severe impacts at advanced life stages [20]. Additionally, innovations in green chemistry and enzyme-mediated degradation are being investigated to accelerate plastic breakdown without generating harmful byproducts [21]. While these approaches are still under development, they offer potential pathways toward reducing the ecological footprint of plastic use and minimizing plastamination.

This hidden crisis demands urgent and coordinated action. Plastamination lies at the nexus of some of the most pressing planetary health challenges, with far-reaching implications for ecosystems and human well-being. The climate cost of plastic production is staggering, generating an estimated 1.8 Gt of CO₂ annually, equivalent to the emissions of approximately 370 million cars, which significantly contributes to global warming and undermines climate mitigation efforts [2]. MPs have also been detected in major rivers, such as the Ganges, Yangtze, and Nile, raising concerns about the quality of freshwater and the health of communities that rely on these vital water sources [22-24].

Beyond environmental and health impacts, plastic pollution imposes staggering economic costs on ecosystems and societies. A 2023 WWF report estimated that the global cost of plastic pollution, including its impacts on ecosystem services, health, waste management, and greenhouse gas emissions, could reach US$7.1 trillion by 2040 if current trends persist [25]. Other analyses suggest that the actual cost of single-use plastics, if externalities were fully priced in, would far exceed their market value. Some estimates suggest they should cost 10 to 100 times more to account for environmental damage, health burdens, and economic loss. Incorporating these hidden costs into plastic pricing through taxes, extended producer responsibility schemes, and international trade regulations could play a transformative role in reducing plastic use and funding mitigation strategies.

Developing and promoting safe, biodegradable materials that do not fragment into harmful MPs/NPs in natural environments is critical. At the same time, comprehensive regulations of plastics must be enforced throughout their entire life cycle, from design and production to use and final disposal. Increased investment in exposome research is essential to map lifelong exposure to micro- and nano-plastics and better understand their health impacts. Furthermore, strong global governance frameworks must be established to integrate environmental sustainability with health equity and intergenerational justice.

Without decisive and coordinated intervention, plastamination will continue to erode ecological resilience, exacerbate health disparities, and undermine the well-being of current and future generations. Tackling this crisis requires a One Health approach that integrates environmental, animal, and human health strategies. We recommend implementing full life cycle regulations for plastic production and disposal, accelerating research on exposure thresholds and biomarkers in humans, investing in sustainable material innovation, and embedding environmental cost accounting into plastic pricing. Public awareness campaigns and individual behavioral shifts, such as reducing the use of single-use plastics and supporting circular economy models, are also essential. Only through a multisectoral, equity-focused response can we mitigate the long-term consequences of plastamination and promote a healthier, more sustainable planet.
